# Neolignan Kadsurenin F Modulates Proteostatic Pathways and Possesses Potent Anti‐Inflammatory Properties

**DOI:** 10.1002/cbdv.202401848

**Published:** 2024-11-23

**Authors:** Zoi Evangelakou, Stefan Schwaiger, Despoina D. Gianniou, Ioannis P. Trougakos, Hermann Stuppner

**Affiliations:** ^1^ Department of Cell Biology and Biophysics Faculty of Biology National and Kapodistrian University of Athens Athens 15784 Greece; ^2^ Institute of Pharmacy/Pharmacognosy University of Innsbruck CCB, Innrain 80–82 6020 Innsbruck Austria

**Keywords:** Anti-inflammatory, *Drosophila melanogaster*, Kadsurenin F, NF-κB pathway, Proteasome inhibitor

## Abstract

Kadsurenin F, a natural neolignan‐type compound, has been described as a constituent of various members of the Lauraceae family such as *Aniba* spp. or *Nectandra* spp., but can also be found in various *Piper* species such as *Piper kadsura* Ohwi (Piperaceae). This species is traditionally used to treat asthma, rheumatic pain, arthritis, and digestive problems. Recently, several studies have highlighted the significant anti‐inflammatory potential of *P. kadsura* extracts and secondary metabolites. Here, we report the isolation of kadsurenin F as an active component of *P. kadsura*. We found that kadsurenin F increases oxidative load and suppresses proteasome functionality in normal diploid human fibroblasts, and after administration in *Drosophila* flies. Moreover, kadsurenin F likely possesses anti‐inflammatory properties, as apart from suppressing proteasome activity, it reversed inflammatory phenotypes and inhibited NO production in RAW 264.7 macrophage cells when administered in parallel with LPS. Our findings suggest that the kadsurenin F scaffold can be used for the development of novel highly bioactive proteasome inhibitors and/or anti‐inflammatory compounds.

## Graphical abstract

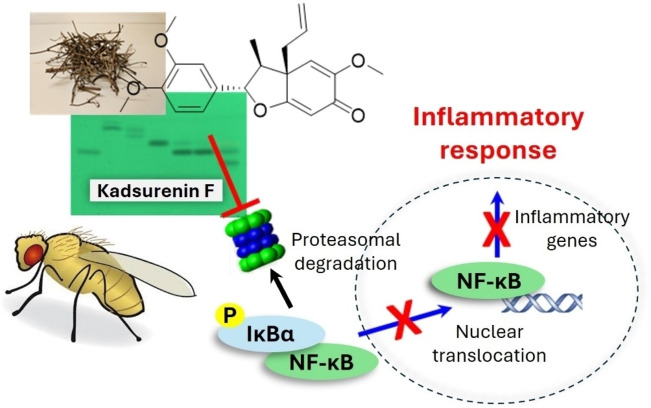

## Introduction

Organisms have developed pathways involved in maintaining the proteome's integrity, collectively called the proteostasis network (PN). Key components of the PN are the protein synthesis and trafficking modules, the molecular chaperones and the two main degradation machineries, namely the Ubiquitin‐Proteasome (UPP) and the Autophagy‐Lysosome (ALP) pathways.[^1^, ^2^] UPP consists of the ubiquitin‐conjugating enzymes and the 26S proteasome and degrades short‐lived polyubiquitinated native proteins and/or non‐functional or misfolded polypeptides.^[3]^ Thus, it can be thought of as the standby degradation mechanism of the cells that facilitates a variety of processes, such as development, immunity, metabolism, signal transduction, cell cycle progression, and cell death.^[3]^ Additional integrated modules of the PN are the regulatory pathways of cellular stress,[^4^, ^5^] that among others include the family of the inducible nuclear factor‐κB (NF‐κB) transcription factors,^[6]^ which regulate a large array of genes involved in different processes of the immune and inflammatory responses.^[7]^ The NF‐κB proteins are normally sequestered in the cytoplasm by inhibitory proteins, including the members of the IκB family and related proteins characterized by ankyrin repeats.^[8]^ The proteasome is an important regulator of NF‐κB signaling, as it participates in the activation of the canonical NF‐κB pathway by degrading its negative regulator IκBα to release the transcriptionally active p50/p65 heterodimer; in the non‐canonical NF‐κB pathway, the proteasome processes the active p52 NF‐κB subunit from the longer precursor p100.[^9^, ^10^] Reactive Oxygen Species (ROS) activate the NF‐κB pathway mainly by modulating the phosphorylation status of IκBα, whereas NF‐κB target genes [particularly c‐Jun N‐terminal kinase (JNK)] attenuate ROS.[^11^, ^12^]

In recent years, the compound class of (dihydro)benzofuran neolignans has been proposed as an interesting source of novel scaffolds for the treatment of inflammation.^[13]^ In this study, we focused on a different subtype, which carries the side chain not on the aromatic ring unit but on the fusion side of the furan ring with the aromatic moiety. Representatives of this compound subclass can be found in different plant families e. g. Lauraceae or Piperaceae. As a biological source of neolignans *Piper kadsura* (Choisy) Ohwi proved to be a rich source.[^14^, ^15^] This species is a perennial vine‐like medicinal plant, which grows mainly in the southeast of China and is widely used for the treatment of rheumatoid arthritis and asthma.^[16]^ Studies during the last 30 years have shown that *P. kadsura* exerts anti‐platelet activating factor (PAF),^[17]^ anti‐inflammatory,^[18]^ antioxidant,^[19]^ and neuroprotective[^15^, ^16^] properties. Several compounds have been isolated from *P. kadsura*, including amide alkaloids, neolignans, terpenes, and cyclohexanes.[^15^, ^16^, ^18^, ^20^] Very recently, constituents of *P. kadsura* were investigated for their ability to suppress NO production in LPS‐stimulated RAW 264.7 cells identifying (*R*)‐futoquinol, kadsurenin F, and (−)‐denudatin B as the most promising candidates, but also with a notable impact on cell viability at the tested concentrations.^[21]^ Interestingly, kadsurenin F is not only known as a constituent of *P. kadsura* but was also isolated some years earlier from the South American Lauraceae species *Aniba burchellii* and *A. affinis*,[^22^, ^23^] or a *Nectandra* species.^[24]^ Here, kadsurenin F isolated from the stems of *P. kadsura* was subjected to a detailed biological and pharmacological investigation using a variety of cell‐based and *in vivo* experimental models.

## Results and Discussion

Lignans and neolignans are secondary plant metabolites originating from oxidative coupling of two C_6_‐C_3_ monomers.^[25]^ Due to different coupling positions, ring opening as well as formation of oxygen‐containing rings and bridges, a wide chemical variability is possible within this compound class resulting in a broad spectrum of possible pharmacological activities. Based on previous studies with derivatives of the (dihydro)benzofuran subtype,^[13]^ we were interested in exploring the pharmacological potential of a different neolignan subtype bearing the allyl‐chain at the annulation position.

**Figure 1:**
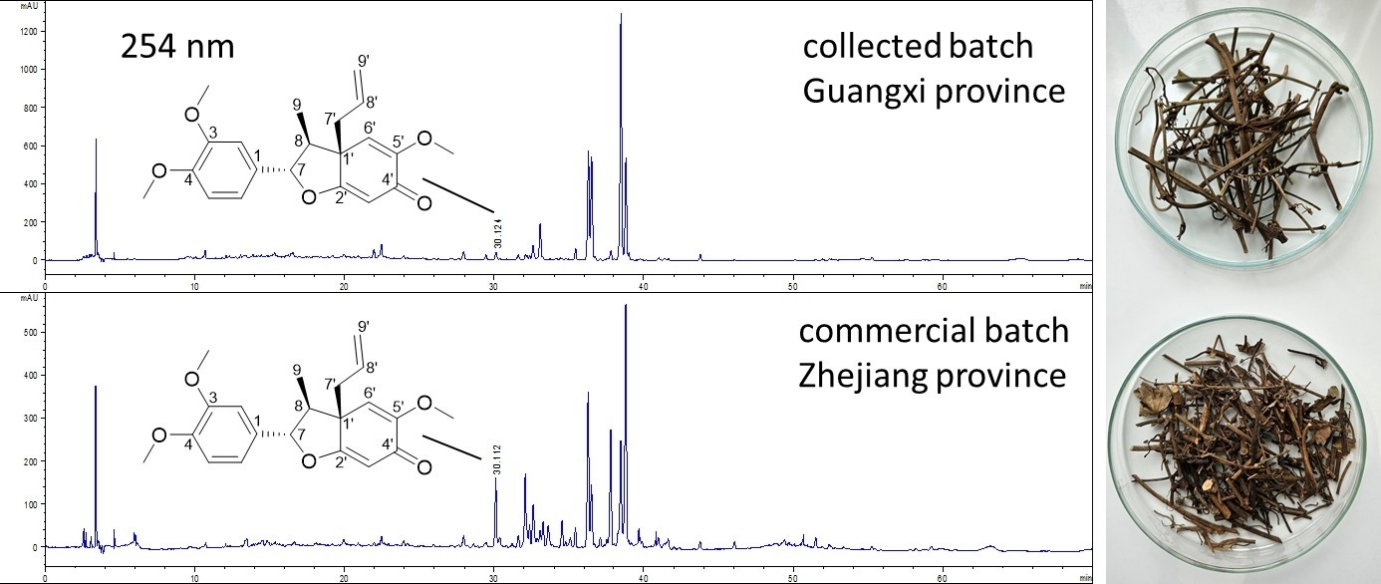
Chromatogram of the HPLC‐DAD analysis of the crude methanolic extract (5 mg/mL methanol) of the stems of *P. kadsura* at 254 nm of the two different plant batches (right part) used for isolation. Marked peak at Rt 30.1 min represents kadsurenin F (chemical structure shown as insert). For experimental conditions and protocols, see Experimental section.

Given that such compounds can be found in different plant families, for this study we focused on a member of the Piperaceae family used in traditional Chinese medicine. The stems of *Piper kadsura* are used in Traditional Chinese Medicine for the treatment of asthma and arthritic conditions.^[18]^ Extracts from this plant have been reported to have anti‐human hepatitis B virus, anti‐platelet activating factor, anti‐insect feeding and anti‐inflammatory activities.[^20^, ^26^, ^27^, ^28^, ^29^] Nevertheless, little is known about the molecular mechanisms underlying its biological activities. Kadsurenin F was therefore isolated from the stems of two different batches of *P. kadsura*, which showed huge quantitative differences in the respective neolignane content (Figure 1).

The pure compound obtained was characterized by LC–MS, 1D and 2D NMR. In addition, optical rotation was measured and an ECD spectrum was recorded to verify not only the relative but also the absolute configuration of the compound.^[23]^ An overview of the experimental data is given in Figures S1 to S9, as well as in Table S1.

**Figure 2:**
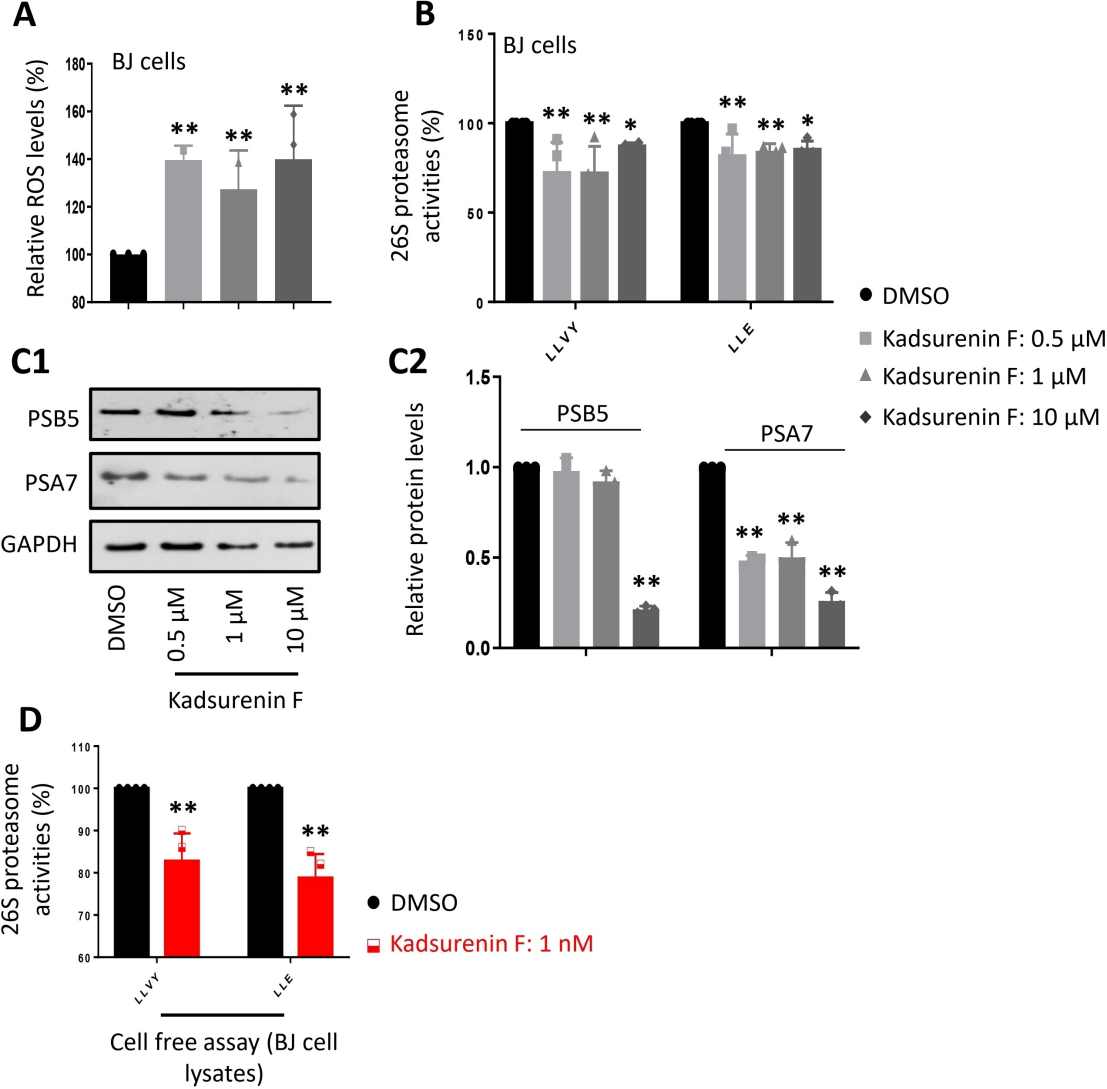
Kadsurenin F suppresses proteasome peptidase activities in skin fibroblasts (BJ cells). (A) Relative (%) ROS levels after treatment for 24 h with the indicated concentrations of kadsurenin F. (B) Relative (%) proteasome peptidase activities (LLVY/CT−L and LLE/C−L) in cells treated with the indicated concentrations of kadsurenin F for 24 h. (C1) Representative immunoblot analysis of the 26S proteasome subunits PSB5 and PSA7 expression levels after cell exposure to the shown concentrations of kadsurenin F for 24 h. (C2) Relative protein quantification of the 26S proteasome subunits PSB5 and PSA7 expression levels following cell exposure to the shown concentrations of kadsurenin F for 24 h (D) Relative (%) proteasome peptidase activities (LLVY/CT−L, and LLE/C−L) in BJ cell lysates incubated with 1 nM kadsurenin F. GAPDH probing (C) was used as a reference for protein input. Control samples (untreated cells) values were set to 100 % in (A, B, D) or 1 in (C2). Bars, ±SD; *p<0.05; **p<0.01.

Since a multitude of the mentioned traditional indications can be drawn back to an imbalance in ROS, the effects of kadsurenin F on cellular proteostatic mechanisms were investigated by evaluation of its impact on cell oxidative load and proteasome functionality. We found that exposure to non‐toxic concentrations of kadsurenin F led to increased oxidative load in BJ cells (Figure 2A) and reduced proteasomal Chymotrypsin‐like (CT−L) and Caspase‐like (C−L) peptidase activities (Figure 2B); this effect paralleled the downregulated expression of the α7 and β5 proteasomal subunits in BJ fibroblasts (Figures 2C1, 2). The effects do not appear to be concentration‐dependent, which may be due to a plateau effect.

Interestingly, addition of the compound directly to BJ cell lysates containing free proteasomes suppressed proteasome activities (Figure 2D), suggesting that kadsurenin F likely acts as a direct binder and therefore as a genuine proteasome inhibitor.

**Figure 3:**
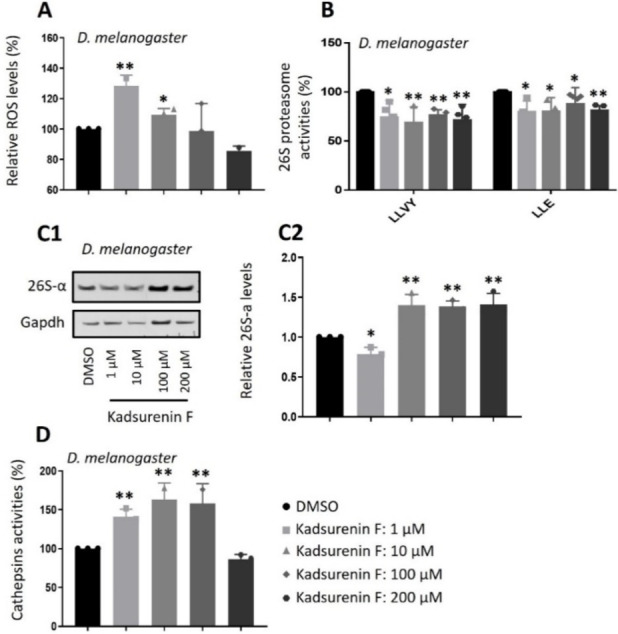
Kadsurenin F acts as a proteasome inhibitor in *Drosophila melanogaster*. (A) Relative (%) ROS levels in *D. melanogaster* flies’ somatic tissues following administration of the indicated concentrations of kadsurenin F for 96 h. (B) Relative (%) proteasome peptidase activities (LLVY/CT−L and LLE/C−L) in *D. melanogaster* flies’ somatic tissues following administration of the indicated concentrations of kadsurenin F for 96 h. (C1) Representative immunoblot analysis of the 26S proteasome subunits α7 (26S‐α) expression levels after flies’ exposure to the shown concentrations of kadsurenin F for 96 h. (C2) Relative protein quantification of the 26S proteasome subunits α7 (26S‐α) expression levels after flies’ exposure to the shown concentrations of kadsurenin F for 96 h (D) Cathepsins peptidase activity in somatic tissues of *D. melanogaster* flies fed with indicated concentrations of kadsurenin F for 96 h. Gapdh probing (C) was used as a reference for protein input. Control values were set to 100 % in (A, B, D) or 1 in (C2). Bars, ±SD; *p<0.05; **p<0.01.

In order to verify those cell‐based findings in a more complex biological context, the administration (see Experimental Section) of kadsurenin F in *Drosophila* flies was selected as an *in vivo* experimental model. Our subsequent studies in *Drosophila* flies revealed that, as found in BJ cells, administration of kadsurenin F in flies increased oxidative load (at 1 and 10 μM) in flies’ tissues (Figure 3A) and mildly suppressed proteasome activities (Figure 3B) leading at higher concentrations to the counteracting induction of proteasomal subunits expression (Figures 3C1, 3). At higher concentrations (100, 200 μM) kadsurenin F may induce cellular stress or toxicity, or even activate alternative molecular pathways, which may then explain the no further increase of ROS (Figure 3A). Notably, administration of kadsurenin F in flies markedly increased the activity of the pro‐survival lysosomal cathepsins activity (Figure 3D). Considering that compounds with weak proteasome inhibitory activity (as shown for kadsurenin F in BJ cells and flies) often possess anti‐inflammatory properties,[^30^, ^31^, ^32^, ^33^, ^34^, ^35^, ^36^], we evaluated kadsurenin F at cell‐based inflammation models using the murine (RAW264.7) cell line.

**Figure 4:**
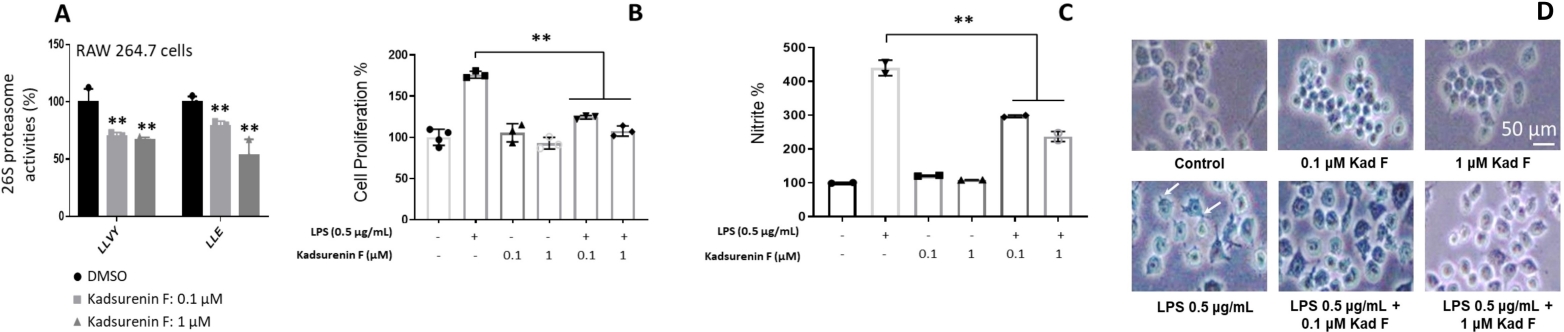
Kadsurenin F exerts anti‐inflammatory properties in LPS‐stimulated mammalian monocytes. (A) Relative (%) proteasome peptidase activities (LLVY/CT−L, and LLE/C−L) in RAW 264.7 cells treated with the indicated concentrations of kadsurenin F for 24 h. (B) Proliferation rate (%) of RAW 264.7 cells treated with the indicated concentrations of kadsurenin F and/or LPS stimulation (0.5 μg/mL) for 24 h. (C) Relative (%) quantification of nitrite (NO) production in RAW 264.7 cells treated with the indicated concentrations of kadsurenin F and/or LPS stimulation (0.5 μg/mL) for 24 h. (D) Representative phase contrast images of morphological changes in RAW 264.7 cells after stimulation with 0.5 μg/mL LPS and treatment with 0.1 or 1 μΜ of kadsurenin F. In (A–C) control values (unstimulated cells) were set to 100 %. Bars, ±SD; *p<0.05; **p<0.01. Bars, in (D) 50 μm.

Our results showed that kadsurenin F did not exhibit significant cytotoxicity against normal human (BJ) (not shown) and murine (RAW264.7) (Figure 4B) cell lines.

We observed that similarly to our previous findings, kadsurenin F reduced dose‐dependently proteasomal Chymotrypsin‐like (CT−L) and Caspase‐like (C−L) peptidase activities in murine RAW264.7 macrophages at concentrations of 100 nM and 1 μM (Figure 4A). Co‐treatment of RAW 264.7 cells with kadsurenin F concentrations and lipopolysaccharide (LPS; an inflammation inducer) resulted in a reduced proliferative ratio (Figure 4B) and decreased nitrate production (Figure 4C) *vs*. sole LPS treatment.

**Figure 5:**
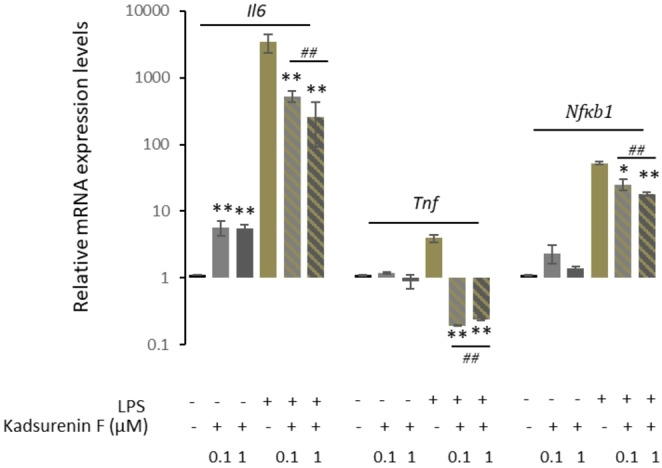
Kadsurenin F alleviates the expression levels of *Il6*, *Tnf* and *Nfkb1* genes in LPS‐stimulated RAW 264.7 macrophage cells. Relative mRNA expression of *Il‐6* and *Tnf* genes, as well as of the *Nfkb1* gene in RAW 264.7 cells treated with the indicated concentrations of kadsurenin F and/or LPS stimulation (0.5 μg/mL) for 24 h. Values in control samples (unstimulated cells) were set to 1; *Actin* gene expression was used as a reference for total RNA input. Bars, ±SD; (*p<0.05; **p<0.01 *vs* control group, and ##*p* <0.01 *vs* LPS group).

Interestingly, examination of the RAW 264.7 cells post‐LPS or ‐LPS/kadsurenin F treatment also revealed morphological alterations. Specifically, untreated control group RAW264.7 cells were roundish, with smooth cell edges having no pseudopodia; on the contrary, cells stimulated with LPS had characteristics of macrophages activation, such as elongated pseudopodia. These morphological changes of the RAW264.7 cells were ameliorated when LPS treatment was combined with kadsurenin F (Figure 4D). Consistently, to the suggested anti‐inflammatory action of kadsurenin F, we found a milder induction of the pro‐inflammatory cytokine *Il6*, as well as of *Tnf* and *Nfκb1* genes in LPS‐kadsurenin F treated cells as compared to solely LPS‐stimulated macrophages RAW 264.7 (Figure 5). The induction of the pro‐inflammatory cytokine *Il6* has a substantial impact on the onset and development of diseases associated with inflammation.^[37]^

## Conclusions

In conclusion, kadsurenin F exhibits mild proteasome inhibitory activity in both cellular systems and at *in vivo* assays. Importantly, it demonstrates anti‐inflammatory properties by effectively reversing inflammatory phenotypes and inhibiting NO production in RAW 264.7 macrophage cells when administered concurrently with LPS stimulation. These findings suggest the compound's potential as a promising anti‐inflammatory agent, not only by modulating proteasome activity but also by attenuating inflammatory responses; nonetheless, further investigation is warranted to fully elucidate its mechanism of action and possible therapeutic implications.

## Experimental Section

### General

High‐performance liquid chromatography with photodiode array and mass spectrometry detection (HPLC‐DAD/MS) experiments were carried out with an Agilent 1260 system coupled with an MS Single Quad detector from Agilent with API electrospray ionization (LC–MSD from Agilent Technologies, Waldbronn, Germany). LC parameters: stationary phase: Phenomenex Gemini 3 μm C18 110 Å, 3.0×250 mm; mobile phase: A=H_2_O +0.1 % formic acid, B=acetonitrile +0.1 % formic acid or H_2_O +0.02 % TFA and pure acetonitrile when used without MS detection; gradient: 0 min: 5 % B; 50 min: 98 % B; 70 min: stop; temp.: 40 °C, flow: 0.4 mL/min inj. vol.: 5 μL, sample conc.: extracts 5 mg/mL; fractions 1 mg/mL in MeOH. MS parameter: split, 1 : 5; ESI, negative mode; spray voltage, 4.5 kV; dry temperature, 320 °C; drying gas flow rate, 12.00 L/min; nebulizer gas, 1294 Torr; mode, scan range: *m/z* 100–1200. One‐ and two‐dimensional NMR experiments were recorded on a Bruker Avance II 600 spectrometer (Bruker) equipped with a cryoprobe operating at 600.19 MHz (^1^H) and 150.91 MHz (^13^C) at 300 K (chemical shifts δ in ppm, coupling constants J in Hz), with deuterated chloroform (CDCl_3_) as solvent. The ECD spectrum was recorded in acetonitrile with the JASCO J‐1500 instrument (JASCO, Tokio, Japan), using quartz cuvettes (d=1 cm) and the optical rotation was measured using a JASCO P‐2000 polarimeter.

### Origin of the Plant Material

The stems of *Piper kadsura* (Piperis kadsurea caulis) were purchased from Plantasia (Gröming, Austria), batch number 920855 (Zhejiang province), 2.7 kg. A second batch (2.0 kg) was collected and identified by Anrui Lou in August 2019 at Ziyuan County, Gulin City, Guangxi province. A voucher specimen (SS 2019–2) is stored at the herbarium of the Institute of Pharmacy/Pharmacognosy, University of Innsbruck.

### Extract Preparation and Compound Isolation

The air‐dried starting materials were kept separately during extract preparation. After milling, the powdered material was exhaustively extracted with methanol (each batch 7×5 L) each time by maceration for 24 h at room temperature and sonication (10 min) prior vacuum filtration through a sinter funnel (porosity number 3). Obtained filtrates were combined and evaporated to complete dryness under reduced pressure by using Büchi Rotavapor (Model R‐215) at 40 °C, yielding 46.31 g for the commercial batch and 175.77 g from the collected material. After HPLC analysis (see Figure 1), both extracts were combined (220 g) and triturated with 500 g silica gel 60 (40–63 μm). The mixture was transferred to a glass column and eluted with mixtures of petroleum ether and acetone (each 500 ml, increase in 5 % steps until 50 % acetone), followed by 500 ml 75 % acetone and 500 ml pure acetone as well as 500 ml acetone methanol (50 %) and 500 ml pure methanol. The eluate was collected in 500 ml round flask in portions of ca. 400 ml and evaporated to dryness using a rotorvapor. HPLC analysis of the fractions obtained (5 mg/ml MeOH) revealed kadsurenin F in fractions 7 to 10. Fraction 7 (6.3228 g) was further separated via Sephadex LH20 column chromatography using a gel bed of 3 cm diameter and 75 cm length. The mobile phase consisted of a mixture of dichloromethane and acetone (85+15 v%). The eluate was collected in portions of 8 ml via a fraction collector and the composition was analyzed by TLC (mobile phase *n*‐hexane and acetone, 1+1). Fractions were combined according to their composition and evaporated to dryness. The same procedure was repeated for the combined fractions 8 to 10 (total 8.6196 g). Evaporated fractions of both separations were analyzed by HPLC and enabled the detection of kadsurenin F in Sephadex fraction 7.3 (tube 21–26; 622.7 mg) and Sephadex fraction 8/9/10.2 (tube 13–17; 648.8 mg). The combined fractions were further purified by NP‐MPLC using a Reveleris X2 system (Grace Germany, Worms, Germany) with a 12 g HP Silica cartridge (20 μm). The sample was introduced via a solid loader as mixture with an equal amount of silica gel, elution was performed with a flow rate of 10 ml /min using gradient elution from 100 % petroleum ether to 100 % methyl *tert*‐butyl ether in 15 min followed by 15 min elution with 100 % methyl *tert*‐butyl ether. The compound eluted between 22 and 26 min with a yield of 178.4 mg. The final separation was performed by Sephadex LH20 column chromatography (1.5 cm diameter, length 65 cm) using methanol as mobile phase resulting in 140.52 mg kadsurenin F with a purity of 97 % according to HPLC‐DAD analysis at 254 and 280 nm. 1D‐ and 2D‐NMR spectra and the corresponding NMR data are available in the Supplementary material (Table S1, Figure S1–S8), as well as the LC–MS‐spectrum in API‐ESI positive mode and an LC‐online UV‐spectrum of the compound. Additionally, an ECD spectrum was recorded in acetonitrile (71 μg/ml) and the obtained spectrum is also shown in the Supplementary material (Figure S9).

### *Drosophila* Lines

Oregon R *Drosophila melanogaster* flies were purchased from Bloomington and used as the wild‐type strain.

### Flies Culture and Exposure to Kadsurenin F

Flies’ stocks were maintained at 24 °C, 60 % relative humidity on a 12 h light: 12 h dark cycle and were fed standard medium (unless otherwise indicated). Kadsurenin F was added to the flies’ culture medium; final concentrations of the compound in the diet were 1, 10, 100, and 200 μM (unless otherwise indicated) and the duration of flies’ exposure to the compound was 3 days (unless otherwise indicated).

### Cell Lines and Cell Culture Conditions

Human newborn foreskin (BJ) and the murine macrophage RAW 264.7 cells were purchased from the American Tissue Culture Collection (ATCC). Cells were cultured in Dulbecco's modified Eagle's medium (ThermoFisher Scientific Inc.), supplemented with 10 % (v/v) fetal bovine serum, 2 mM glutamine, and 1 % non‐essential amino acids, in a humidified incubator at 5 % CO_2_ and 37 °C. Cell treatment with the kadsurenin F compound was performed (unless otherwise indicated) for 24 h.

### Cell Survival Assay for RAW 264.7and BJ Cells

The effect of kadsurenin F on the viability of RAW 264.7 and BJ cells was examined using the MTT assay. Briefly, cells were plated in flat‐bottomed 96‐well microplates and the next day were incubated with different concentrations of kadsurenin F and/or lipopolysaccharide (LPS) for 24 h. Afterwards, the medium was replaced by 3‐(4,5‐dimethylthiazol‐2‐yl)‐2,5‐diphenyltetrazolium bromide (MTT, Sigma‐Aldrich) dissolved at a final concentration of 1 mg/ml in serum‐free, phenol red‐free medium. The formed formazan crystals were then dissolved by isopropanol and the absorbance of the solution was measured at 570 nm wavelength. Survival of control cells was arbitrarily set to 100 %.

### Measurement of Proteasome Enzymatic Activities

Skin fibroblast cells (BJ), RAW264.7 macrophages or isolated flies’ somatic tissues were lysed on ice using buffers suitable for the isolation of 26S proteasomes (0.2 % Nonidet P‐40, 5 mM ATP, 10 % glycerol, 20 mM KCl, 1 mM EDTA, 1 mM dithiothreitol and 20 mM Tris, pH 7.6). Lysates were cleared by centrifugation at 19,000× g (4 °C) and 20 μg of proteins were immediately used to determine the main proteasome proteolytic activities [chymotrypsin‐like (CT−L/LLVY) and caspase‐like (C−L/LLE)]. Activities were measured by recording the hydrolysis of the fluorogenic peptides Suc−Leu−Leu−Val−Tyr−AMC and Z−Leu−Leu−Glu−AMC respectively. Fluorescence was measured using a VersaFluor^TM^ Fluorometer System (Bio‐Rad Laboratories, Hercules, CA, USA) at excitation and emission wavelengths of 350 and 440 nm, respectively. Fluorescence intensity was normalized to the total protein level per sample and expressed as the relative percentage *vs*. control (DMSO); in experiments using flies’ tissues equal numbers of male and female flies were used. Each sample was prepared in duplicate.

### Measurement of Reactive Oxygen Species (ROS)

To determine ROS levels, cultured cells or flies’ somatic tissues were collected in PBS and incubated in CM‐H_2_DCFDA dye (Invitrogen, Carlsbad, CA, USA) for 30 min at 25 °C in the dark. Following centrifugation and dye removal, tissues were incubated in PBS for 10 min at 24 °C to allow cellular esterases to hydrolyze the acetoxymethyl ester or acetate groups and render the dye responsive to oxidation. Samples were washed in PBS, lysed in Nonidet P‐40 lysis buffer (1 % Nonidet P‐40, 150 mM NaCl, and 50 mM Tris, pH 8.0), and cleared by centrifugation at 19,000× g for 10 min at 4 °C. The supernatant was diluted 1 : 4 (v/v) in ddH_2_O, and fluorescent dichlorodihydrofluorescein was measured using a VersaFluor Fluorometer System (BioRad Laboratories, Hercules, CA, USA) at excitation 490 nm/emission 520 nm. Negative controls were either unstained tissues incubated with PBS buffer alone to detect autofluorescence or cell‐free mixtures of dye and buffers.

### Measurement of Cathepsins’ Enzymatic Activities in Flies’ Tissues

Dissected somatic tissues of *D. melanogaster* flies were lysed on ice in 1 mM dithiothreitol and 50 mM Tris, pH 4.0, and the lysates were cleared at 14,000× g for 20 min at 4 °C. After the protein content was determined using a Bradford assay (BioRad), 20 mg of protein were incubated in the reaction buffer (50 mM sodium acetate, 8 mM cysteine hydrochloride, 1 mM EDTA, pH 4.0) containing the substrate‐FRAMC (Enzo Life Sciences) for 30 min at 37 °C. The fluorescence was measured (Versa Fluor TM Fluorometer System) at excitation and emission wavelengths of 350 and 440 nm, respectively.

### Measurement of Nitric Oxide (NO) Production

The RAW 264.7 cells were seeded at a density of 5×10^5^ cells/well in 24‐well plates and incubated for 24 h at 37 °C and 5 % CO_2_. Different concentrations of kadsurenin F were prepared in FBS‐free DMEM and after 1 h treatment, cells were stimulated with 0.5 μg/mL of LPS. The production of NO was quantified by measuring the released NO metabolites (nitrates and nitrites) with Griess reagent (Sigma‐Aldrich). After a 24 h exposure, the culture medium samples were collected and made cell‐free by centrifugation. The medium was incubated with the same volume of Griess reagent at room temperature (RT) for 15 min before measuring absorbance at 540 nm in a microplate reader.

### Preparation of Cell Extracts and Immunoblotting Analysis

Cells or isolated tissues were lysed on ice in NP‐40 lysis buffer (150 mM NaCl, 1 % NP‐40, 50 mM Tris pH 8.0) containing protease and phosphatase inhibitors and lysates were cleared by centrifugation at 19 000× g (4 °C; 10 min). The total protein content of each lysate was measured by the Bradford assay (Bio‐Rad). For immunoblotting equal total protein (μg) per sample were separated by SDS‐PAGE and blotted onto a nitrocellulose membrane (Immobilion‐P, Millipore, Eschborn, Germany); primary and horseradish peroxidase‐conjugated secondary antibodies were applied for 1 h at room temperature and immunoblots were developed using an enhanced chemiluminescence reagent kit (Santa Cruz Biotechnology).

### Antibodies Used

Primary antibodies against Prosα7 (sc‐100456) and Prosβ5 (sc‐55009) human proteasome subunits; the 26S‐α (sc‐65755) *Drosophila* proteasome and GAPDH (sc‐25778), as well as the horseradish peroxidase‐conjugated secondary antibodies, were purchased from Santa Cruz Biotechnology.

### RNA Isolation and Quantitative Real Time PCR (q‐RT‐PCR) Analysis

Total RNA was isolated using the NucleoZOL RNA Isolation Reagent (Macherey–Nagel, Düren, Germany) and quantified with BioSpec‐nano spectrophotometer (Shimadzu Inc.). Subsequently, cDNA synthesis and qRT‐PCR were performed using the FastGene Scriptase II cDNA Synthesis 5 × Ready‐Mix (NIPPON Genetics EUROPE, GmbH, Düren, Germany) and the HOT FIREPol® EvaGreen® qPCR Mix Plus (08–36–00001, Solis BioDyne, Tartu, Estonia), respectively. Primers were designed using the primer‐BLAST tool (http://www.ncbi.nlm.nih.gov/tools/primer‐blast; accessed on 8 January 2018). The sequences of *Tnf* primers were 5’‐CGAGTGACAAGCCTGTAGCC‐3’ and 5’‐CTTTGAGATCCATGCCGTTGG‐3’; of *Il6* 5’‐GACAAAGCCAGAGTCCTTCAGA‐3’ and 5’‐AGGAGAGCATTGGAAATTGGGG‐3’; of *Nfkb1* 5’‐GGATACTGAACAATGCCTTCCG‐3’ and 5’‐TGCCTGGATCACTTCAATGGC‐3’, and of *Actin* 5’‐GGCTGTATTCCCCTCCATCG‐3’ and 5’‐CCAGTTGGTAACAATGCCATGT‐3’.

## Author Contributions

Author Contribution: HS and IPT designed and supervised the study; ZE, SS, and DDG conducted experiments. All authors interpreted the data; have read and have agreed to the final version of the manuscript.

## Conflict of Interests

The authors declare no conflict of interest.

1

## Supporting information

As a service to our authors and readers, this journal provides supporting information supplied by the authors. Such materials are peer reviewed and may be re‐organized for online delivery, but are not copy‐edited or typeset. Technical support issues arising from supporting information (other than missing files) should be addressed to the authors.

Supporting Information

## Data Availability

The data that support the findings of this study are available in the supplementary material of this article.
