# Supplementary material for: Neolignan Kadsurenin F Modulates Proteostatic Pathways and Possesses Potent Anti‐Inflammatory Properties
Source: Chem Biodivers. 2024 Nov 23;22(3):e202401848. doi: 10.1002/cbdv.202401848 (PMC11908775; doi:10.1002/cbdv.202401848)

Chemistry & Biodiversity

Supporting Information

Neolignan Kadsurenin F Modulates Proteostatic Pathways and Possesses Potent Anti-Inflammatory Properties

Zoi Evangelakou, Stefan Schwaiger,* Despoina D. Gianniou, Ioannis P. Trougakos, and Hermann Stuppner*

Supplementary Material

Table of contents

Figure S1. HPLC-MS spectrum of kadsurenin F

Figure S2. HPLC-online UV-spectrum of kadsurenin F

Figure S3. ^{1}H-NMR spectrum of kadsurenin F

Figure-S3-1. Magnification of the aromatic region of the ^{1}H-NMR-spectrum of kadsurenin F

Figure S4. ^{13}C-NMR spectrum of kadsurenin F

Figure S5. ^{1}H,^{1}H-COSY-NMR spectrum of kadsurenin F

Figure S6. HSQC-NMR spectrum of kadsurenin F

Figure S7. HMBC-NMR spectrum of kadsurenin F

Figure S8. NOESY-NMR spectrum of kadsurenin F

Figure S9. ECD spectrum of kadsurenin F

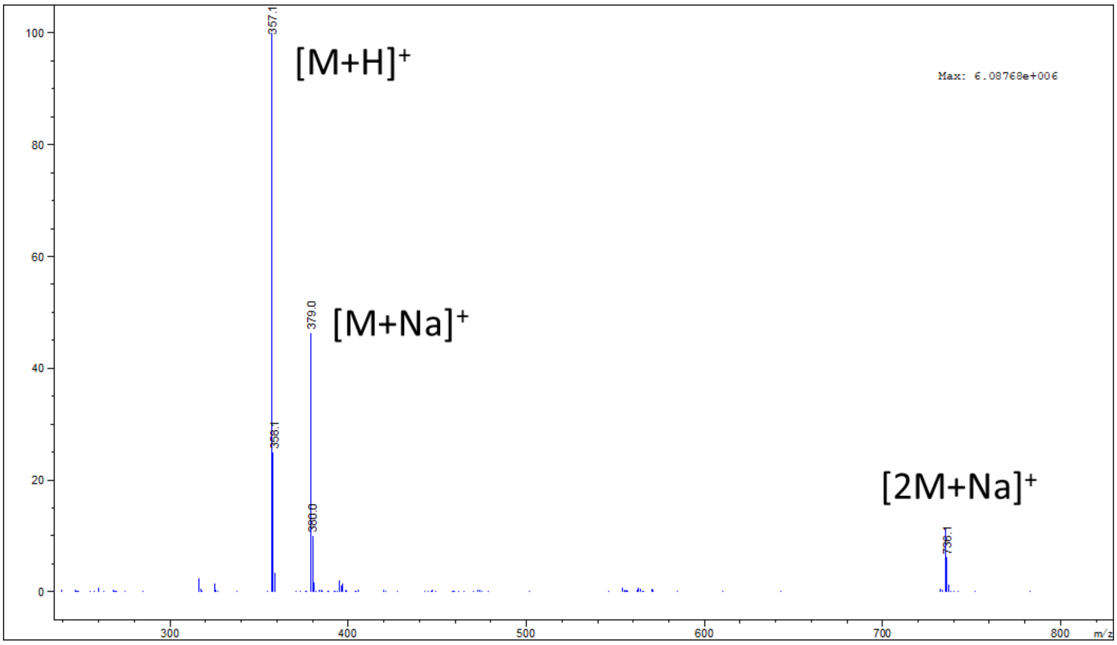

Figure S1. HPLC-MS spectrum of kadsurenin F; API-ESI, positive mode. For analytical conditions, see Experimental Section main article.

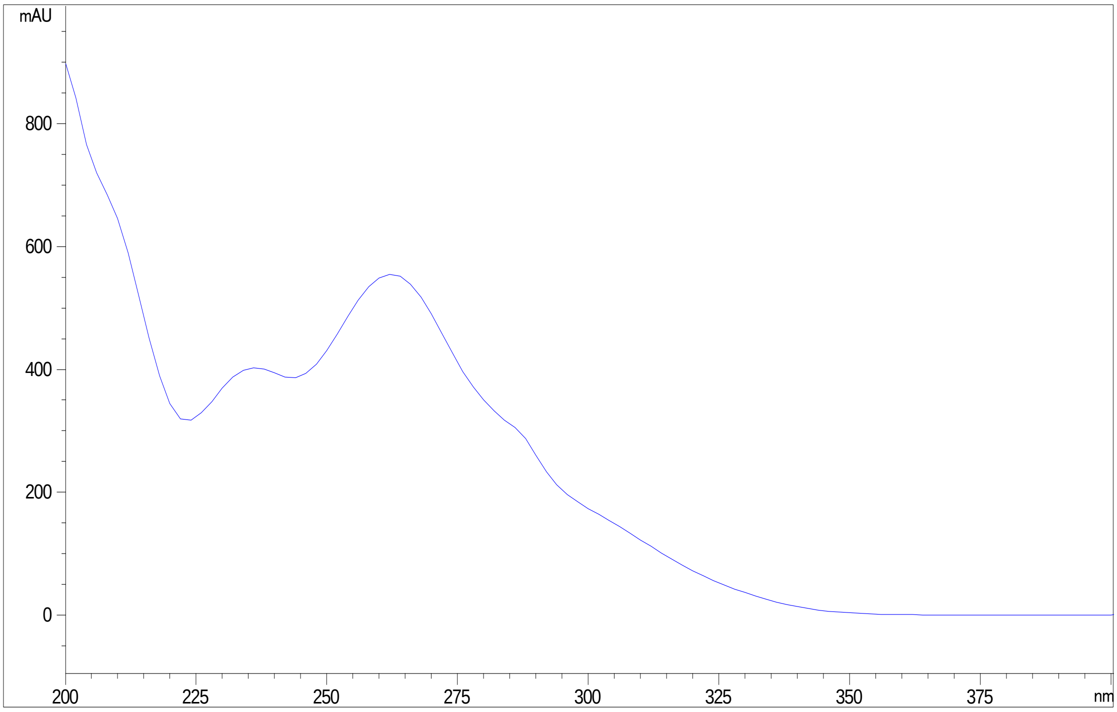

Figure S2. HPLC-online UV-spectrum of kadsurenin F. For analytical conditions, see Experimental Section main article.

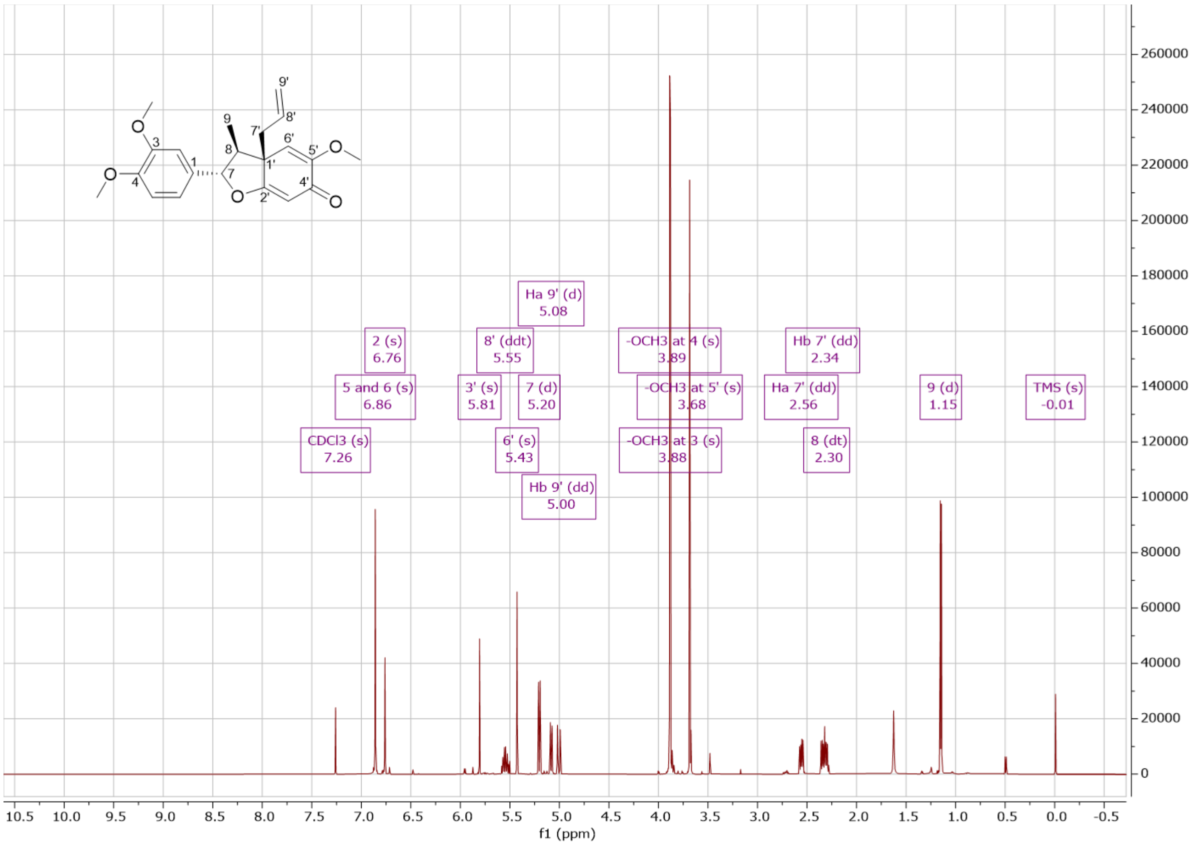

Figure S3. ^{1}H-NMR spectrum of kadsurenin F in $CDCl_3$ at 600.17 MHz.

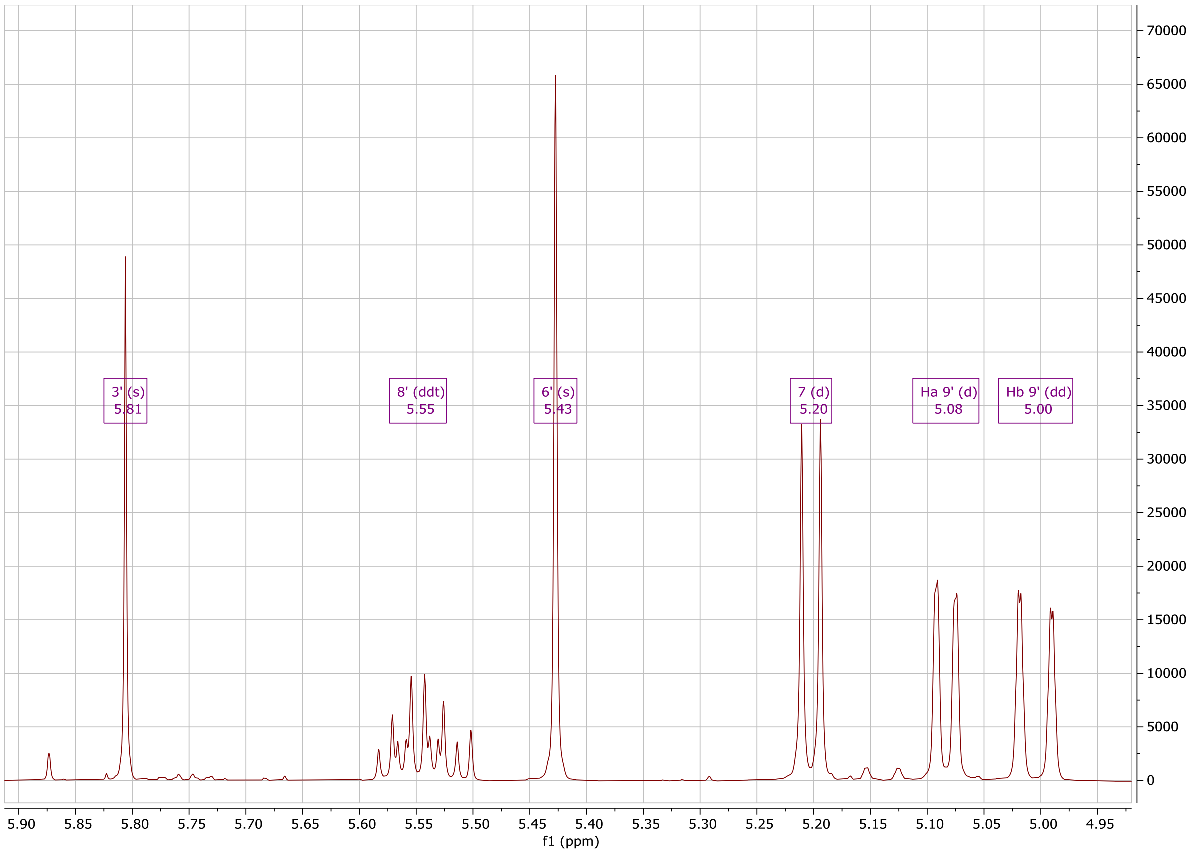

Figure-S3-1. Magnification of the aromatic region of the ^{1}H-NMR-spectrum of kadsurenin F in $CDCl_3$ at 600.17 MHz

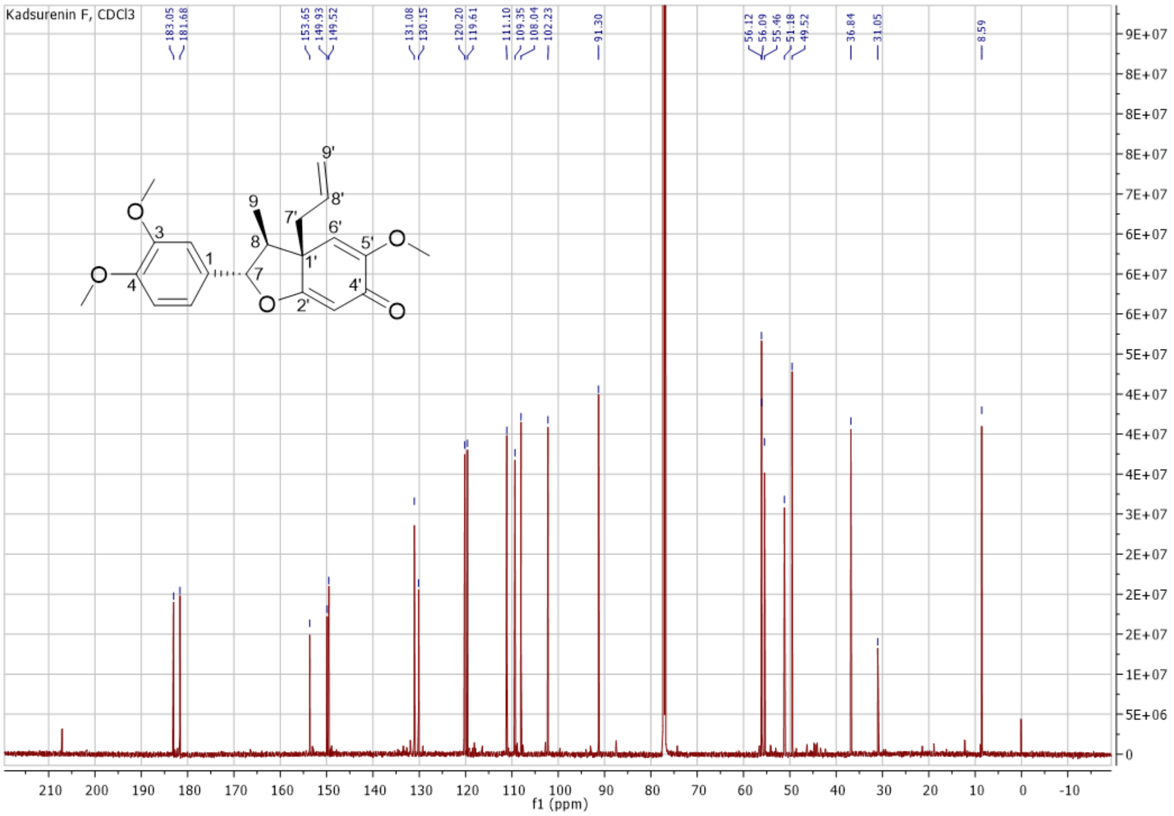

Figure S4. ^{13}C-NMR spectrum of kadsurenin F in $CDCl_3$ at 150.91 MHz.

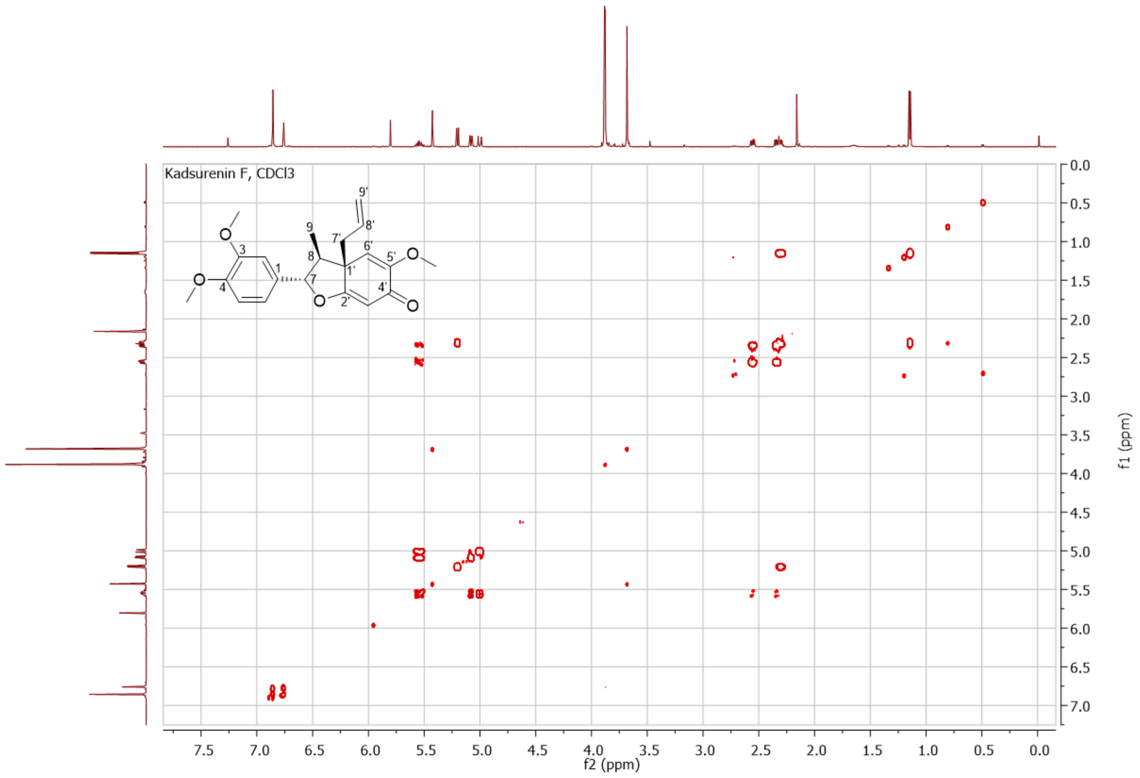

Figure S5. ^{1}H,^{1}H-COSY-NMR spectrum of kadsurenin F in $CDCl_3$ at 600.17 MHz.

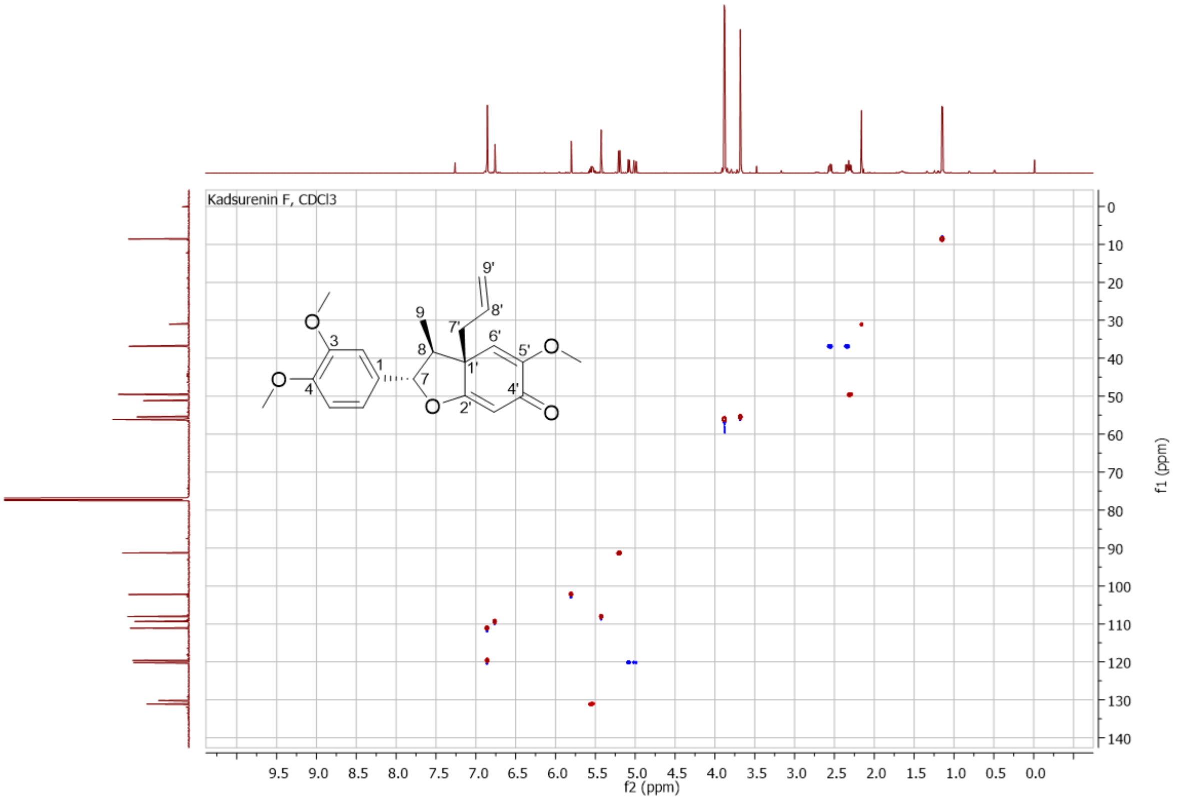

Figure S6. HSQC-NMR spectrum of kadsurenin F in $CDCl_3$ at 600.17/150.91 MHz.

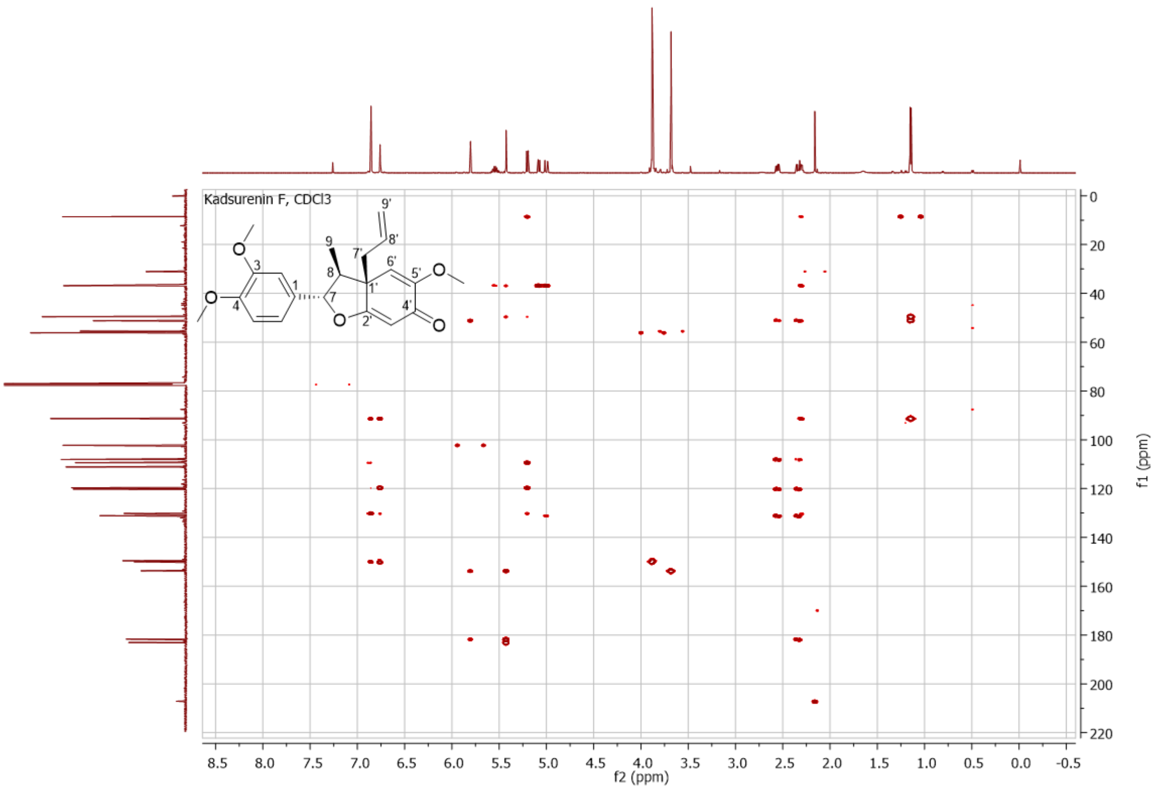

Figure S7. HMBC-NMR spectrum of kadsurenin F in $CDCl_3$ at 600.17/150.91 MHz.

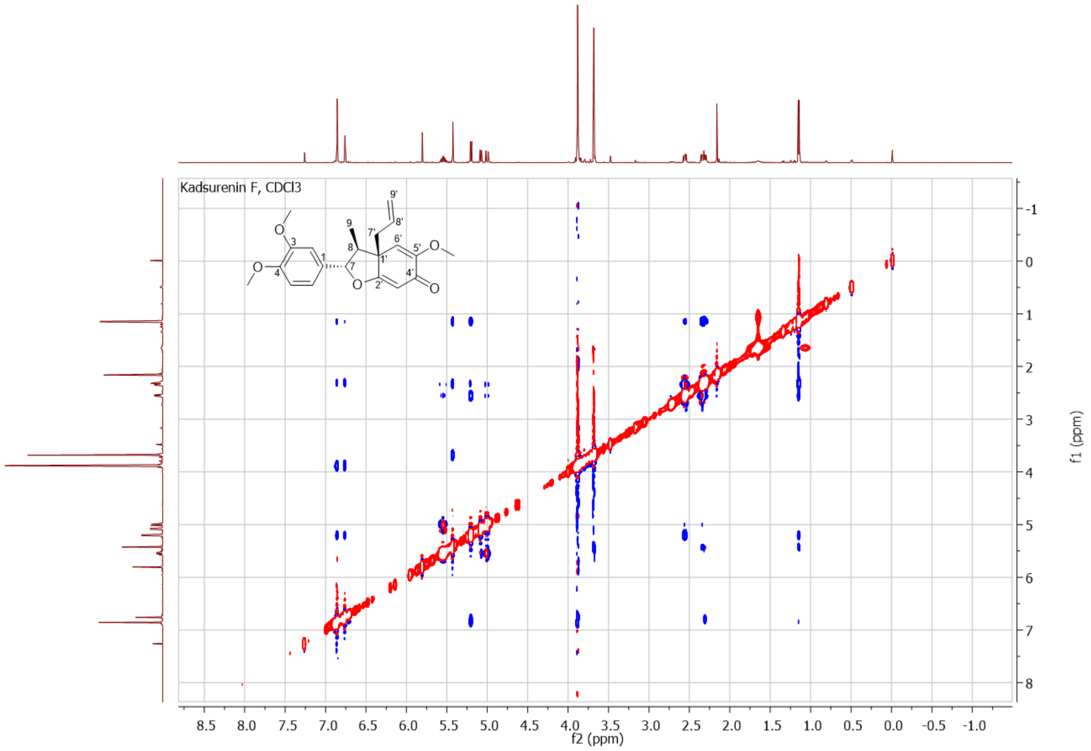

Figure S8. NOESY-NMR spectrum of kadsurenin F in $CDCl_3$ at 600.17 MHz.

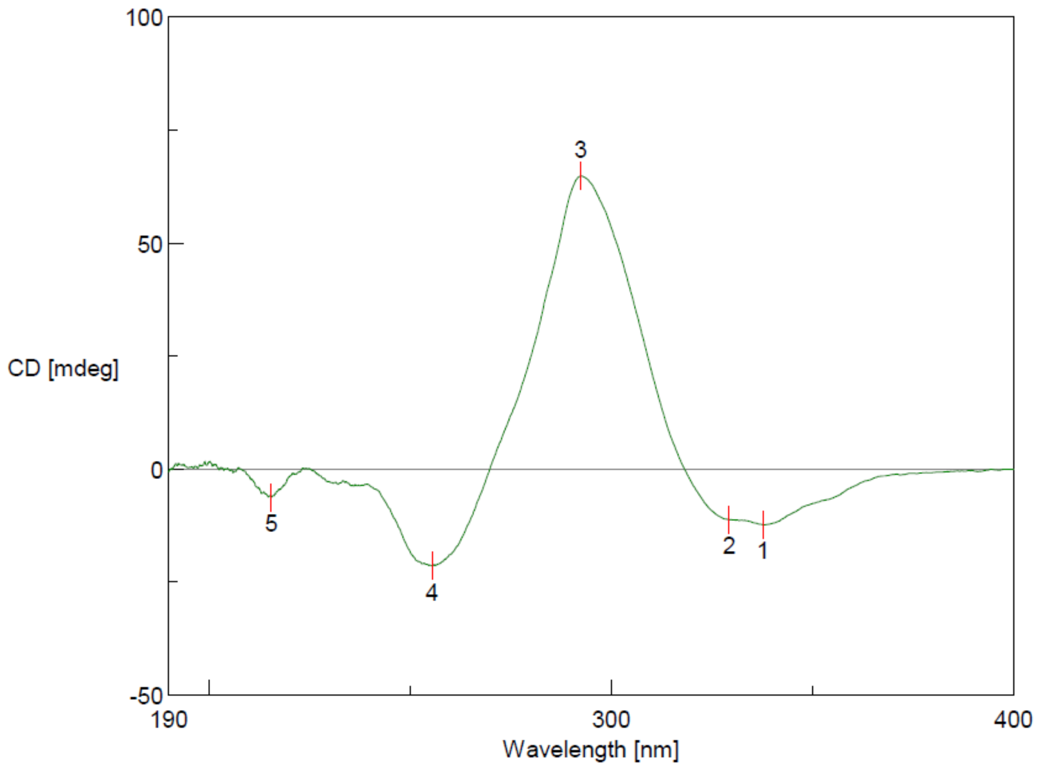

Figure S9. ECD spectrum of kadsurenin F in acetonitrile (71 µg/mL, corrected). Observed maxima, wavelength [nm]/CD [mdeg]:337.7/-12.4329; 329.3/-11.3102; 292.4/64.8482; 255.5/-21.5166; 215.5/-6.33683.

Table 1. Experimental NMR data of kadsurenin F.

Position	***^{1}H-NMR data at 600.19 MHz in $CDCl_3$, J in Hz***	***^{13}C-NMR data at 150.91 MHz in $CDCl_3$***
1	---	130.15
2	6.76 s, 1H	109.35
3	---	149.52
4	---	149.93
5	6.86 br s, 1H	111.10
6	6.86 br s, 1H	119.61
7	5.20 d, J = 10.0 Hz, 1H	91.30
8	2.30 dt, J = 10.0, 6.7 Hz, 1H	49.52
9	1.15 d, J = 6.9 Hz, 3H	8.59
1'	---	51.18
2'	---	181.68
3'	5.81 s, 1H	102.23
4'	---	183.05
5'	---	153.65
6'	5.43 s, 1H	108.04
7'	H_a 2.56 dd, J = 13.4, 7.1 Hz<br>H_b 2.34 dd, J = 13.2, 7.2 Hz	36.84
8'	5.55 ddt, J = 17.2, 10.2, 7.2 Hz, 1H	131.08
9'	H_a 5.08 br d, J = 10.1 Hz<br>H_b 5.00 dd, J = 17.0, 1.6 Hz	120.20
-OCH3 at 3	3.879 s, 3H	56.08*
-OCH3 at 4	3.885 s, 3H	56.12*
-OCH3 at 5'	3.68 s, 3H	55.46
****might be exchanged***		

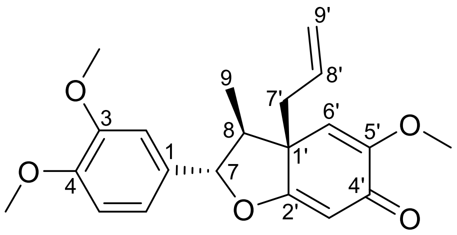

Supplement: Supplementary file 1 — Supporting Information [file CBDV-22-e202401848-s001.pdf]
